# Salivary Metabolomics Discloses Metabolite Signatures of Oral Leukoplakia with and Without Dysplasia

**DOI:** 10.3390/ijms26136519

**Published:** 2025-07-07

**Authors:** Elena Ferrari, Rita Antonelli, Mariana Gallo, Marco Meleti, Giacomo Setti, Adele Mucci, Valeria Righi, Anna Gambini, Cristina Magnoni, Alberto Spisni, Thelma A. Pertinhez

**Affiliations:** 1 Laboratory of Biochemistry and Metabolomics, Department of Medicine and Surgery, University of Parma, 43125 Parma, Italy; elena.ferrari@unipr.it (E.F.); alberto.spisni@unipr.it (A.S.); 2 Centro Universitario Odontoiatria, University of Parma, Via Gramsci 14, 43126 Parma, Italy; rita.antonelli@unipr.it (R.A.); marco.meleti@unipr.it (M.M.); 3 Dentistry and Oral-Maxillofacial Surgery Unit, University of Modena and Reggio Emilia, 41125 Modena, Italy; giacomo.setti@unimore.it; 4 Department of Chemical and Geological Sciences, University of Modena and Reggio Emilia, 41125 Modena, Italy; adele.mucci@unimore.it (A.M.); anna.gambini@unimore.it (A.G.); 5 Department for Life Quality Studies, University of Bologna, 47921 Rimini, Italy; valeria.righi2@unibo.it; 6 Dermatology Unit, Department of Surgical, Medical, Dental & Morphological Sciences with Interest Transplant, Oncological & Regenerative Medicine, University of Modena and Reggio Emilia, 41125 Modena, Italy; cristina.magnoni@unimore.it

**Keywords:** salivary metabolomics, salivary diagnostics, leukoplakia, dysplasia, malignant transformation

## Abstract

Leukoplakia is a condition marked by white patches on the inner surfaces of the oral cavity. Its potential to progress to oral squamous cell carcinoma underscores the need for effective screening and early diagnosis procedures. We employed NMR-based salivary and tissue metabolomics to identify potential biomarkers for leukoplakia and dysplastic leukoplakia. Univariate and multivariate methods were used to evaluate the NMR-derived metabolite concentrations. The salivary metabolite profile of leukoplakia exhibited specific alterations compared to healthy controls. These metabolic changes were more pronounced in cases of dysplastic lesions. Multivariate ROC curve analysis, based on a selection of salivary metabolites, ascribed high diagnostic accuracy to the models that discriminate between dysplastic and healthy cases. However, NMR analysis of tissue biopsies was ineffective in extracting metabolic signatures to differentiate between lesional, peri-lesional, and healthy tissues. Our pilot study employing a metabolomics-based approach led to the development of salivary models that represent a complementary strategy for clinically detecting leukoplakia. However, larger-scale validation is required to fully evaluate their diagnostic potential and to effectively stratify leukoplakia patients according to dysplasia status.

## 1. Introduction

Saliva is a biofluid increasingly employed in diagnostics and clinical research, not only for its easy, safe, and non-invasive sampling protocol but also for its translational value. It is currently used in the diagnosis of various diseases, including Alzheimer’s, diabetes, pancreatitis, adrenal cortex diseases, and gastric and breast cancer [1,2], and, more broadly, it provides precious hints to refine the comprehension of a person’s health status. The variety of metabolites present in saliva has stimulated clinical research in oral and systemic diseases, leading to the identification of disease-related biomarkers and the assessment of metabolite profiles under homeostasis [3,4,5,6,7,8,9]. Notably, the direct contact of saliva with oral lesions suggests that the description of its metabolic profile may result in a sensitive screening tool for the detection of biomarkers of oral potentially malignant disorders [10,11].

Oral leukoplakia is characterized by white lesions on the oral mucosa, which are generally asymptomatic. This condition is particularly worrying as it is the most common oral precancerous condition, with a current global prevalence of 4.11% [12,13]. A systematic review and meta-analysis conducted between 1996 and 2022 revealed that the estimated prevalence of oral leukoplakia was higher among males, individuals aged over 60 years, smokers, and alcohol consumers [14]. Considering its potential to progress to oral squamous cell carcinoma (OSCC), it is essential to implement effective screening and early diagnosis procedures [14,15].

The diagnosis of leukoplakia requires a clinical examination, followed by a biopsy. The latter is performed to confirm or correct the clinical diagnosis of leukoplakia and to verify the presence and grade of dysplasia. In clinical practice, the presence and grade of epithelial dysplasia have demonstrated prognostic utility in stratifying the risk for malignant transformation (MT) [13,16]. In a prospective observational study of localized oral leukoplakia, oral epithelial dysplasia was identified as the most significant predictor of MT. Integrating DNA ploidy into the model enhanced its predictive power [17]. The precision of clinicians in diagnosing leukoplakia and assessing the risk of MT mostly relies on their professional skills and expertise [18].

The development of non-invasive diagnostic methods and the validation of potential biomarkers are primary objectives of oral leukoplakia research [15]. To date, several studies have employed salivary metabolomics in an attempt to identify biomarkers associated with that condition and MT risk. Mass spectrometry studies have explored the salivary metabolite profile in OSCC and oral leukoplakia [19,20,21]. However, the clinical utility of the observed metabolite signatures to anticipate MT and/or to enhance the prognosis of OSCC remains to be assessed.

The objective of the present pilot study is to identify salivary metabolites that can assist in differentiating patients diagnosed with oral leukoplakia from healthy controls and patients with mild dysplastic leukoplakia from those with non-dysplastic leukoplakia.

Chemometrics, uni- and multivariate analyses, were employed to analyze NMR-derived metabolites’ concentrations. The combination of specific salivary features enabled the identification of metabolic signatures that classify patients with dysplastic and non-dysplastic leukoplakia. Based on the exploratory approach adopted, we propose a promising biomarker profile that, after further validation, could be used for preventive clinical screenings and oral leukoplakia surveillance.

## 2. Results

### 2.1. Demographic Data of Study Participants

Out of the 38 volunteers who participated in the present study, 26 subjects were diagnosed with oral leukoplakia, either with or without dysplasia. The diagnoses were made based on clinical examination and further confirmed by histopathological analysis. Two subjects exhibited non-homogeneous leukoplakia lesions, while all others exhibited homogeneous lesions. Table 1 presents the demographic data of the study population. Among the 26 patients diagnosed with leukoplakia, 19 were over 60 years old. The highest percentage of smokers was found in the mild dysplastic leukoplakia (DLK) group, followed by the non-dysplastic *leukoplakia* (NDLK) and healthy control (HC) groups. The highest incidence of dysplastic leukoplakia lesions was found in the tongue mucosa, in 7 out of 13 cases (Table 2). Notably, two of the dysplastic leukoplakia lesions occurred in the youngest participants in the study, who were females aged 22 and 24 years.

Five NDLK patients presented two lesions. In four cases, both lesions were observed in the same mucosal region. Two DLK patients had two lesions, and one exhibited three lesions. In a single case, the lesions manifested in the same mucosal region.

### 2.2. Metabolite Composition of Salivary Samples

Metabolomic analysis based on ^1^H-NMR spectroscopy enabled the identification and quantification of 72 salivary metabolites in patients with NDLK and DLK, and 71 in healthy subjects. The three groups displayed comparable metabolite profiles, except for inosine, which was absent in the HC.

The comprehensive metabolites’ datasets of NDLK, DLK, and HC samples can be classified into the following main groups: amino acids and their derivatives, simple carbohydrates, lactate, pyruvate, compounds of bacterial origin, and intermediates of the tricarboxylic acid (TCA) cycle, lipid and phospholipid metabolism, and purine and pyrimidine metabolism.

### 2.3. Statistical Analysis of the Metabolite Datasets

**Figure 1:**
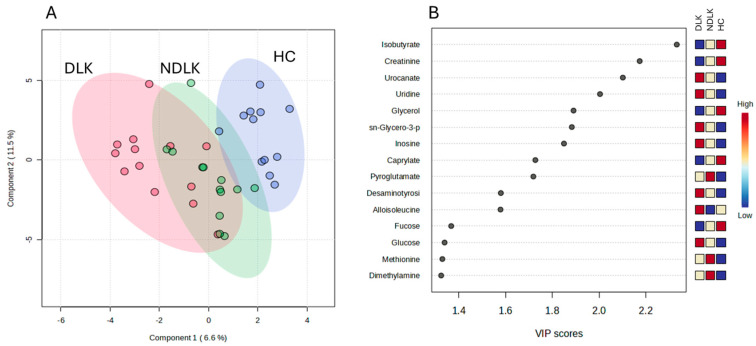
Partial least squares discriminant analysis (PLS-DA) scores plot of salivary metabolite datasets from the NDLK, DLK, and HC groups (**A**). The coloured ellipses represent the 95% confidence region of each cluster. Variable’s importance in projection (VIP) plot (**B**) ranks the most discriminative metabolites resulting from PLS-DA component 1. A metabolite with a higher VIP score discriminates more effectively between the groups. The blue and red boxes illustrate the relative metabolite abundance in the specified groups.

The partial least squares discriminant analysis (PLS-DA) model (Figure 1) obtained with the three metabolite concentration datasets effectively separated the metabolomes of the patients and the HC groups. Notably, the analysis revealed that the metabolomes of the NDLK patients showed a greater degree of overlap with those of the DLK patients than with those of the HC subjects.

**Figure 2:**
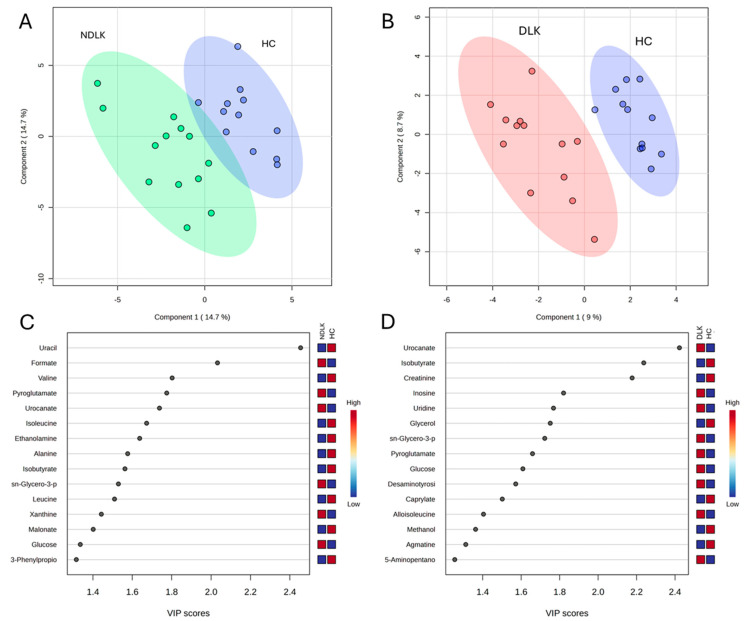
Partial least squares discriminant analysis (PLS-DA) scores plot of the salivary metabolite datasets from the NDLK, DLK and HC groups. (**A**) NDLK vs. HC, (**B**) DLK vs. HC. The coloured ellipses represent the 95% confidence region of each cluster. PLS-DA score plots are flanked by their corresponding variable’s importance in projection (VIP) plots, (**C**,**D**), which rank the most discriminative metabolites resulting from PLS-DA component 1. A metabolite with a higher VIP score discriminates more effectively between the two groups. The blue and red boxes illustrate the relative abundance of metabolites in the specified groups.

To highlight the metabolic differences between pathological and healthy states, PLS-DA models were constructed for the metabolomes of NDLK and HC, as well as DLK and HC (Figure 2A,B). The variable’s importance in projection (VIP) score plots in Figure 2C,D correspond to these PLS-DA models and indicate the metabolites that contribute more to group separation by component 1. Interestingly, both models identified urocanate, isobutyrate, *sn*-glycero-3-phosphocholine, and pyroglutamate. The PLS-DA model of the DLK and NDLK metabolomes was unable to effectively discriminate between the two pathological conditions (Figure S1A). The metabolites most significantly contributing to their group separation are listed in the VIP score plot in Figure S1B.

**Figure 3:**
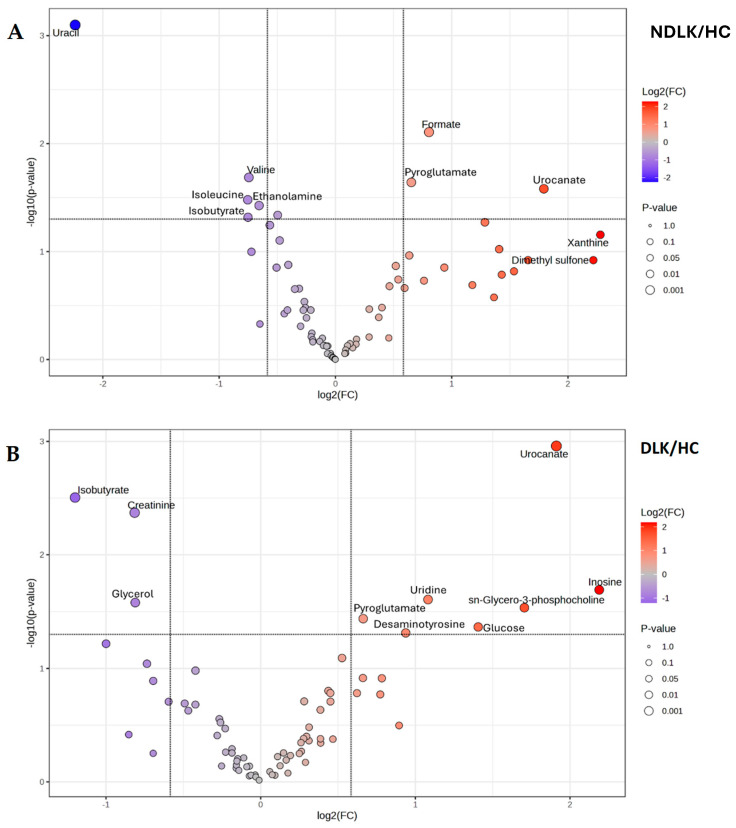
Volcano plot analysis of the salivary metabolite datasets from (**A**) the patients with leukoplakia vs. healthy controls (NDLK/HC) and (**B**) the patients with dysplastic leukoplakia vs. healthy controls (DLK/HC). For each metabolite compared, the plots combine its fold change (log_2_ (FC) on the *x*-axis) and −log_10_ *p*-value from the *t*-test, on the *y*-axis. Metabolites that satisfy the *p* < 0.05 and |FC| > 1.5 are considered discriminant and represented by coloured circles in the upper left and right quadrants.

The metabolite concentrations measured in the saliva of each subject group were averaged to obtain the corresponding metabolite profiles. A visual comparison of the metabolites’ levels is provided in Figure S2, where a heatmap highlights the differences in the metabolite profiles of NDLK, DLK, and HC. The volcano plots in Figure 3A,B result from a comparative analysis of the metabolite profiles between the NDLK and DLK conditions and the HC, respectively. For each metabolite in the two conditions, the plots combine its fold change (FC, on the *x*-axis) and *p*-value (from the *t*-test, on the *y*-axis). The metabolites exhibiting the largest and most significant concentration differences (|FC| > 1.5, *p* < 0.05) are located within the upper left and right quadrants of the volcano plots.

In the DLK group, a greater number of significant metabolites exhibited an increase in concentration compared to HC than in the NDLK group (Figure 3). In the NDLK/HC plot, uracil is associated with the highest statistical significance and negative FC (in log_2_ scale). A comparable degree of significance was observed for creatinine and isobutyrate in the DLK/HC plot, although with a lower |FC| (Figure 3A). The metabolites urocanate, isobutyrate, and pyroglutamate were significant in the NDLK/HC and DLK/HC comparisons but not in the DLK/NDLK comparison (Figure S1C).

As expected, all the most discriminant metabolites identified in the volcano analyses (Figure 3 and Figure S1C) also exhibit high VIP scores in the corresponding PLS-DA models (Figure 2C,D and Figure S1B).

To account for potential confounding factors arising from cohort heterogeneity (Table 1), we applied a linear model with covariate adjustments for age, smoking status, and sex. The most significant metabolites identified by these adjusted linear models are presented in Table 3. It is worth noting that only metabolites detected in more than 50% of the samples were included in this analysis; consequently, inosine was excluded. Importantly, the metabolites identified as significant after covariate adjustment were also found to be significant in analyses without covariate adjustment, further supporting the robustness and validity of our findings.

### 2.4. Receiver Operating Characteristic (ROC) Curve Analysis

A preliminary ROC analysis was conducted to determine the efficacy of each metabolite to discriminate between the NDLK, DLK, and HC groups. For the univariate ROC analysis of NDLK vs. HC, DLK vs. HC, and DLK vs. NDLK, we employed the MetaboAnalyst platform (www.metaboanalyst.ca, accessed on 1 July 2025) to calculate the t-statistics and Area Under the Curve (AUC) for each metabolite detected. For the multivariate ROC analyses, we selected metabolites with significant concentration differences between groups (adjusted *p*-value < 0.05) and univariate AUC values greater than 0.80, assuming the combination of these metabolites would enhance their discriminative potential. The multivariate ROC curves corresponding to NDLK vs. HC, DLK vs. HC, and DLK vs. NDLK classifications are presented in Figure S3. As expected, the combination of significant metabolites improves the diagnostic performance of the models, as shown by the higher AUCs obtained in the multivariate than in the single metabolite ROC curve analyses.

### 2.5. NMR and Statistical Analysis of Intact Oral Mucosa Biopsies

Concurrently with saliva collection, samples of lesion and perilesional tissue from the NDLK and DLK patients, as well as samples of healthy tissue from the healthy subjects, were used for NMR measurements. Biopsies from two subjects were discarded due to poor spectra quality, reducing the number of leukoplakia samples to 24.

The analysis of ^1^H-NMR spectra led to the identification of 42 metabolites. Both univariate (ANOVA) and multivariate analyses (Principal Components Analysis, PLS-DA and orthogonal PLS-DA) were performed on the normalized tissue NMR spectra to highlight differences between the five sample groups: HC, DLK, NDLK, perilesional tissues of DLK, and perilesional tissues of NDLK. No appreciable differences in the metabolic profiles were found between DLK vs. perilesional DLK, NDLK vs. perilesional NDLK, DLK vs. NDLK, DLK + NDLK vs. HC (Figure S4).

## 3. Discussion

The increasing number of clinically focused metabolomic studies offers great potential for the development of diagnostic applications based on multivariate statistical analysis [22,23,24]. In this scenario, there is an increasing interest in the use of saliva as a diagnostic matrix [10,25,26,27,28,29,30,31,32]. The integration of lab-on-a-chip devices and point-of-care testing is rendering saliva-based diagnostics more practical. These tools hold considerable potential for the real-time monitoring of health parameters and the development of personalized medicine. The current literature emphasizes the need for standardization of sample collection and processing and for larger, multicenter trials to be conducted.

The study of Martins-Chaves et al. [33] investigated the tissue metabolic differences between malignant transformed oral leukoplakia and non-transformed oral leukoplakia. Using untargeted high-performance liquid chromatography–mass spectrometry, researchers identified several molecular features, primarily lipids, that distinguish the two forms of leukoplakia. Additionally, the review by Mohd Faizal et al. [34] highlighted citrate, pyruvate, and glutamate as potential metabolic biomarkers for oral leukoplakia. 

The present ^1^H-NMR-based metabolomic study of saliva and tissue samples aims to identify metabolic biomarkers that can discriminate between healthy subjects and patients with leukoplakia, as well as between patients with dysplastic and non-dysplastic leukoplakia. The analytical approach identified over 70 polar metabolites in all saliva samples, defining a set of metabolites that could be assessed as potential diagnostic factors. An interesting case is inosine, a unique metabolite detected in the saliva in a subset of DLK and NDLK subjects, but not in the saliva of healthy controls. This finding is consistent with previous metabolomic studies from our Lab [8,9].

PLS-DA analysis revealed that the separation between the metabolomes of NDLK and HC and between the DLK and HC was more pronounced than the separation between the DLK and NDLK (Figure 2A,B and Figure S1A). The metabolites urocanate, isobutyrate, *sn*-glycero-3-phosphocholine, and pyroglutamate were found to be discriminative in both the NDLK and DLK vs. HC assessments, but with different VIP scores (Figure 2C,D). Urocanate and *sn*-glycero-3-phosphocholine levels were lowest in the HC profile and increased progressively in the NDLK and DLK profiles. Instead, isobutyrate concentration was highest in HC and exhibited a progressive decrease in the NDLK and DLK profiles (Figure 1B). Based on these findings, including the peculiar distribution pattern of inosine, we conclude that the salivary profile of leukoplakia reveals specific metabolic alterations compared to healthy controls, which are exacerbated in dysplastic conditions.

As indicated in the demographic data of the participants, this study considers several potential confounding factors, including age, sex, and smoking status. A further analysis, conducted to execute significance testing while adjusting for these covariates, validated some of the VIP metabolites previously identified through PLS-DA and volcano plot analyses (Figure 2 and Figure 3, and Table 3 and Table S1).

Multivariate ROC curve analysis was performed by selecting metabolites with an AUC > 0.8 in univariate ROC analysis and an adjusted *p*-value < 0.05 to generate predictive models for subject classification (Table 3). For the DLK vs. HC model, a high diagnostic accuracy (AUC = 0.92) was obtained by combining the effects of creatinine, urocanate, and *sn*-glycero-3-phosphocholine. It is worth noting that these metabolites also appear as the most discriminant ones between the two groups in the volcano plot analysis (Figure 3B). The NDLK vs. HC univariate model based exclusively on formate, one of the most discriminant metabolites in the VIP and volcano plot analyses (Figure 2C and Figure 3A), achieved an AUC = 0.81.

It is worth noting that urocanate, involved in histidine metabolism, plays a pivotal role in differentiating the DLK and HC metabolomes, with a less pronounced role in distinguishing the NDLK and HC metabolomes (VIP scores of approximately 2.4 and 1.8, respectively; Figure 2C,D). According to Hart et al., urocanate levels in skin, urine, and faecal samples may be considered metabolic biomarkers of diverse cutaneous diseases [35]. Kitabatake et al. observed increased urocanate levels in DLK samples relative to NDLK samples, with a median fold change of 1.25 [36]. Additionally, a study on salivary metabolic profiling for diagnosing OSCC revealed that urocanate levels increased significantly between premalignant lesions and OSCC [37].

We observed that the nucleosides inosine and uridine, which are involved in purine and pyrimidine metabolism, were more abundant in the DLK metabolomes than in the HC ones (Figure 2B). Song et al. listed inosine and uridine (and other nucleosides) among the significantly changed metabolites between premalignant lesions and OSCC [37]. In a metabolomic study comparing South American patients diagnosed with OSCC to healthy subjects, inosine was found exclusively in the saliva of OSCC patients. It was proposed as a biomarker (AUC = 0.92) [38]. In line with this study, we did not detect inosine in healthy controls but validated its presence in several dysplastic leukoplakia and leukoplakia metabolomes.

The metabolic signature that better predicted the association with the NDLK vs. DLK group consisted of the branched-chain amino acids (BCAA) and uridine, although with reduced accuracy (AUC = 0.81) compared to the other models (Figure S3). Kitabatake et al. also pursued our research focus in a metabolomics cross-sectional study based on capillary electrophoresis mass spectroscopy [36]. In our experimental conditions, altered concentrations of leucine, valine, and isoleucine, suggesting altered BCAA metabolism, contributed to the DLK vs. NDLK classification model (Table 3, Figure S3C), in accordance with the limited separation between the DLK and NDLK metabolomes determined by PLS-DA (Figure S1A). According to Wei et al. [21], the lower concentration of valine, leucine, and isoleucine in the saliva of OSCC patients compared to healthy controls is presumably due to their increased utilization by the TCA cycle in cancer cells. In the present study, BCAA levels are reduced in the NDLK profile compared to the HC profile but increased in the DLK profile (Figure S2).

^1^H-NMR biopsy analysis was less effective than saliva analysis in extracting metabolic signatures to differentiate between lesional, peri-lesional, and healthy tissues. The lesions and the peri-lesioned tissues revealed heterogeneity with different cell types and associated tissues. In addition, the HC tissue samples were obtained from the gingiva, while the biopsied lesions were collected from eight different sites within the oral cavity (Table 2). The metabolic differences in the biopsies were likely masked by the variability in the sampling site, which included different types of mucosa, and by the presence of NMR signals from residual anesthetic, which forced us to exclude the corresponding spectral regions.

These initial salivary results prompt progress in this direction. The preliminary AUC values indicate potential but require validation of independent, well-matched cohorts before clinical application. The availability of a larger number of metabolomes will contribute to the validation of the proposed biomarkers. The selection of an adequate number of healthy and pathological subjects should be complemented by a more standardized assessment of the participants’ demographic characteristics and risk factors.

According to Sarode et al. [39], a longitudinal study could better reveal any progressive changes in salivary metabolites of leukoplakia patients and witness the eventual MT of LK lesions. However, ethical issues may arise in follow-up studies of potentially malignant diseases, justifying the retrospective design of the most-published studies on leukoplakia [16].

We recognize that the predictive model developed to distinguish between DLK and NDLK patients has limited diagnostic accuracy (Table 3). Nevertheless, integrating salivary metabolomics with oral microbiome profiling, as well as other omics approaches, is expected to enhance the precision and utility of diagnostic models for complex oral diseases. Metabolomics offers insight into biochemical changes associated with disease conditions, while microbiome profiling reveals shifts in microbial communities that may contribute to or reflect pathological processes. Their integration may uncover associations between specific microbial species and metabolites, thereby improving the specificity and sensitivity of diagnostic models. Several studies have investigated the impact of the oral microbiota on oral leukoplakia, aiming to justify its progression to malignancy [40,41,42]. The analysis conducted by Pietrobon et al. revealed a decrease in bacterial richness and diversity in the oral cavity of LK patients compared to HC, accompanied by an increase in anaerobes [42]. Combining these approaches with other omics layers, such as transcriptomics and proteomics, could help identify cross-domain biomarkers useful for stratifying patients according to leukoplakia severity.

## 4. Materials and Methods

### 4.1. Ethical Statement and Study Population

The protocol of this pilot study was approved by the Ethics Committee of “Area Vasta Emilia Nord” (AVEN) (protocol number: 38/2017/TESS/AUOMO-509/2019/TESS/UNIPR); EC Approval Date: 11 September 2018 (prot. AOU 0022735/18 13 September 2018). The study adhered to the ethical standards outlined in the Declaration of Helsinki. Written informed consent was obtained from all eligible subjects before their study enrolment.

The present observational study involved thirty-eight subjects recruited at the Centro Universitario di Odontoiatria of the University of Parma, Parma, Italy, and at the Unità di Dermatologia e Odontoiatria of the Azienda Ospedaliero-Universitaria Policlinico di Modena, Modena, Italy.

The subjects selected for this study are allocated to three categories: patients diagnosed with leukoplakia (NDLK), patients with mild dysplastic leukoplakia (DLK), and healthy controls (HC).

The healthy participants underwent the surgical removal of the third molar at the clinical facilities. Under the protocol approved by the Ethics Committee, the selection of these control subjects allowed for a biopsy to be taken near the extraction site and evaluated in a clinical study of tissue metabolomics. As a result, the age (and gender) of the healthy controls could not be matched to that of the patients, most of whom were older.

The participant selection, which spanned a 3-year period (2021–2023), excluded subjects with hyposalivation (whole saliva flow < 1 mL/5 min) or other oral mucosal or systemic diseases (such as diabetes, hypertension, and cardiovascular and cerebrovascular diseases) and those who had received any adjuvant therapy, such as chemotherapy or radiotherapy, before saliva collection.

### 4.2. Diagnosis of Leukoplakia

The diagnostic process involved a clinician/dentist and a pathologist. The provisional diagnosis was based on a clinical evaluation conducted by a dentist at the clinical facilities. This evaluation included visual inspection and manual palpation, which were used to assess the lesion’s colour, texture, and margins. Based on these clinical data, the lesions were classified as “homogeneous” and “non-homogeneous”, as reported for the first time by Pindborg et al. [43]. Excisional biopsy specimens (n = 26) were obtained from patients with a preliminary diagnosis of leukoplakia. The histopathological analysis allowed us to exclude other oral diseases, assess the presence and degree of epithelial dysplasia, and rule out any oral carcinoma. The process resulted in a definitive clinicopathological diagnosis of oral leukoplakia, with or without dysplasia. The specimens were classified as epithelial dysplastic (DLK, n = 13) or non-dysplastic (NDLK, n = 13).

### 4.3. Saliva Collection and NMR Sample Preparation

Saliva was collected before local anaesthesia and biopsy sampling or molar extraction. The protocol for saliva collection was previously described [8]. Briefly, a sample of unstimulated whole saliva (WS) was collected from each participant. Before collection, subjects were instructed to rinse their mouths with water for one minute. During the collection stage (5–15 min), saliva samples were transferred to a tube containing sodium azide (0.5% final concentration) and maintained on ice until a volume of approximately 2 mL was achieved. These samples were immediately frozen at −80 °C until use.

Each frozen sample was thawed on ice for NMR sample preparation and centrifuged at 15,000× *g* for 10 min at 4 °C. This step was carried out to remove eukaryotic and prokaryotic cells, cellular debris, and mucins, as described by Quartieri et al. [44]. To deplete proteins that may interfere with the quantification of metabolites by NMR, 1 mL of each saliva supernatant was ultra-filtered using Amicon Ultra-4 Centrifugal filters (3000 MWCO, Merck Millipore, Burlington, MA, USA) at 4000× *g* and 10 °C for 60 min. The ultra-filtered supernatants were freeze-dried and resuspended in 600 µL of 25 mM phosphate buffer at pH 7.4, containing 1.45 mM 3-trimethylsilyl propanoic acid (TSP), which served as a quantitative standard, and 5% D_2_O for the solvent signal lock.

#### ^1^H-NMR Spectra Acquisition and Analysis

One-dimensional ^1^H-NMR spectra of saliva samples were acquired at 25 °C using a JEOL 600 MHz ECZ600R spectrometer (JEOL Inc., Tokyo, Japan) as previously described [45]. The spectra were processed and analyzed with the Chenomx NMR Suite 9.0 software (Chenomx Inc., Edmonton, AB, Canada), with zero-filling to 256 K points and a line broadening of 0.5 Hz. A metabolite concentration profile was generated for each study participant. Metabolites with concentrations falling below the quantification limit of the analytical method employed in this study (1 µM) were excluded from further analysis [44].

### 4.4. Statistics on Metabolite Concentrations

Multivariate statistical analysis was conducted on the target metabolites using MetaboAnalyst 6.0 (https://www.metaboanalyst.ca, accessed on 20 October 2024) [46].

To reduce systematic variation and to enhance the performance of downstream statistical analysis, metabolite concentration data were normalized by the median concentration across the experimental samples and auto-scaled (mean-centred and divided by the standard deviation of each variable) before analysis. The missing values (2.2%) were replaced by 1/5 of the minimum positive values of their corresponding variables.

Partial least squares discriminant analysis (PLS-DA) was employed as a supervised multivariate statistical technique. The results were visualized as two-dimensional score plots, and metabolites with high variable importance in projection (VIP) scores were identified. The leave-one-out cross-validation parameters are presented in Table S1. Heatmap analysis, performed using the Pearson distance measure and the “complete clustering” method, provided an intuitive visualization of the differences in metabolite concentrations (Figure S2).

Volcano plots were generated using a combination of fold change (log_2_(FC)) and *p*-value (−log_10_ *p*-value) of the metabolite concentrations compared. A positive log_2_(FC) indicates an increase in concentration, while a negative log_2_ (FC) indicates a decrease when comparing two datasets.

An analysis with covariate adjustment was performed via the linear models with covariate adjustments (Limma) tool in the Metaboanalyst platform to conduct significance *t*-testing while accounting for covariates. The metadata table included age, sex, smoking status, and diagnosis as covariates. Age was treated as a continuous variable, while sex and smoking status were categorical variables.

Potential leukoplakia biomarkers were evaluated by ROC curve analysis. ROC models were built using variable selection (univariate AUC > 0.8) and the PLS-DA algorithm with 2 latent variables, via the Metaboanalyst platform. The cross-validations for DLK vs. HC and NLDK vs. DLK ROC models are presented in the Supplementary Materials (Figure S3). The area under the curve was used to compare the diagnostic accuracy of the selected metabolites.

### 4.5. Mucosa Sample Collection and Preparation for HR-MAS Measurements

To minimize anatomical heterogeneity and pre-analytical variability, all biopsies were collected according to a single standard operating procedure developed jointly by two senior oral surgeons (M.M. and G.S.). For each patient, two spatially matched specimens were obtained with a 4 mm punch or scalpel: (i) a central lesional core, excised from the visually thickest portion of the plaque, and (ii) a peri-lesional sample taken ≥ 5 mm beyond the macroscopic margin within clinically healthy mucosa. This pairwise design allows every patient to serve as their internal control, while preserving local micro-environmental factors (vascularization, microbiota, mechanical stress).

From healthy controls, a single fragment of normal oral mucosa was removed during routine third-molar extraction, either adjacent gingiva or vestibular mucosa.

Each HC, DLK, or NDLK specimen was divided into two portions: one fixed in buffered 10% formalin for routine histology, and the other placed in a sterile tube and snap-frozen in liquid nitrogen. Cryovials were bar-coded in situ and transferred on dry ice to a –80 °C freezer; the cold chain was maintained during all transport.

Before HR-MAS NMR, samples were kept on dry ice during weighing, rotor loading, and locking (total handling time < 5 min), thereby preventing any thaw–freeze cycles. Histology on the formalin-fixed halves confirmed epithelial integrity, and in peri-lesional samples, the absence of dysplasia or a significant inflammatory infiltrate.

This workflow, encompassing anatomical matching, rapid metabolic arrest, and strict cold-chain control, was designed to ensure that any lack of discriminative signals in the HR-MAS NMR dataset reflects genuine biochemical similarity rather than sampling artefacts.

Each frozen biopsy and control tissue was weighed (1–25 mg) and introduced into a zirconia rotor (12 or 50 µL capacity, depending on the sample amount), followed by the addition of 10 µL of D_2_O (99.9%).

#### NMR Data Collection and Analysis

Water-suppressed spin-echo (Carr–Purcell–Meiboom–Gill (CPMG), cpmgpr sequence in Bruker library) ^1^H NMR spectra of biopsies were acquired with an NMR Bruker Avance III HD 600 MHz spectrometer, equipped with a ^1^H, ^13^C, ^31^P high-resolution magic-angle spinning (HR-MAS) probe and a Bruker cooling unit, working at 600.13 MHz on ^1^H. All experiments were conducted at a 4 kHz spin rate and 5 °C to slow tissue degradation. CPMG spectra were transformed with 1 Hz line broadening, manually phased, baseline corrected, aligned, and binned (0.002 ppm) with the MNova software package (MestReNova, ver. 11 Mestrelab Research S. L., Santiago de Compostela, Spain). Biopsies from two subjects were discarded due to poor spectra quality.

Some samples were contaminated with the anesthetics, articaine, and mepivacaine. Their corresponding spectral regions were excluded from the analysis together with the residual water signal and spinning side bands (1.5–1.9 ppm, 1.95–2.3 ppm, 2.75–3 ppm, 3.8–3.9 ppm, 4.2–4.5 ppm, 4.7–5.4 ppm, 5.50–5.75 ppm, 6.02–6.50 ppm, 6.55–6.85 ppm, 6.95–7.3 ppm, 7.45–8.15 ppm). The chemical shift scale was calibrated using the doublet of alanine set at 1.48 ppm. Each spectrum was normalized to the total area. The areas of selected signals from 26 out of the 42 identified metabolites were estimated by deconvolution through the MNova Line Fitting routine on the 64k transformed normalized spectra. Univariate and multivariate statistical analyses were conducted using Metaboanalyst 6.0 (https://www.metaboanalyst.ca [46], accessed on 1 July 2025).

## 5. Conclusions

This pilot study is one of the first investigations to assess the salivary metabolome composition of oral leukoplakia with and without dysplasia. Multivariate ROC analysis based on specific metabolite signatures produced well-performing binary classifiers, achieving high accuracy in discriminating between patients diagnosed with dysplastic leukoplakia and healthy subjects. Indeed, the salivary metabolite signatures, validated with an independent, well-matched cohort, could potentially aid in the diagnosis of dysplastic leukoplakia based on clinical and histopathologic criteria.

We identified specific metabolites (urocanate, *sn*-glycero-3-phosphocholine, and isobutyrate) showing a progressive change in concentration from healthy to leukoplakia and dysplastic leukoplakia. It can, therefore, be suggested that changes in their concentration may indicate an early indicator of the risk of MT. We acknowledge that these findings are preliminary. Should our developed models be validated in a larger study with well-matched cohorts, our metabolomic approach to leukoplakia diagnosis could serve as a useful complement to current clinical detection methods. We further hypothesize that this approach may assist in stratifying patients based on the presence or absence of dysplasia.

## Figures and Tables

**Table 1:** Demographics of the enrolled subjects.

	Mean Age (yr) ± SD ^1^		Smokers % (n)
Study Subjects	Female	Male	
**Healthy Subjects**	29.4 ± 13.6 (n = 7)	38.8 ± 14.5 (n = 5)	25.0 (n = 3)
**Non-dysplastic LK Patients** Homogeneous LK Non-homogeneous LK	63.0 ± 9.6 (n = 6) 62.7 ± 10.5 (n = 6) (n = 0)	66.9 ± 7.3 (n = 7) 67.8 ± 9.8 (n = 5) 66.0 ± 0.0 (n = 2)	38.5 (n = 5)
**Dysplastic LK Patients** Homogeneous LK Non-homogeneous LK	46.5 ± 21.5 (n = 6) 46.5 ± 21.5 (n = 6) (n = 0)	66.0 ± 9.0 (n = 7) 66.0 ± 9.0 (n = 7) (n = 0)	61.5 (n = 8)

^1^ Mean age at the time of biopsy.

**Table 2:** Lesion sites observed in the leukoplakia patients.

	Number of Cases	
Mucosal Lesion Site	NDLK	DLK
Hard palate	1	-
Cheek	4	2
Alveolar	2	-
Gingiva ^1^	2	2
Lip	2	-
Tongue	1	7
Edentulous saddle	1	1
Retromolar trigon	-	1

^1^ Mandibular gingiva in NDLK; maxillary and mandibular gingiva in DLK.

**Table 3:** Potential leukoplakia biomarkers: ROC analysis based on selected salivary metabolites adjusted by covariates.

					Model	
	Selected Metabolites	*p*-Value	*p*-Value adj	|Log_2_FC|	Univariate AUC	Multivariate AUC
NDLK vs. HC	Formate	0.0129	0.0335	1.212	0.81	0.81 ^a^
DLK vs. HC	Creatinine Urocanate *sn*-glycero-3-phosphocholine	0.0067 0.0790 0.0180	0.0720 0.0399 0.0335	1.421 1.057 1.501	0.83 0.83 0.80	0.92
DLK vs. NDLK	Valine Isoleucine Uridine Leucine	0.0059 0.0267 0.0166 0.0374	0.0047 0.0171 0.0314 0.0221	1.122 0.947 0.854 0.909	0.87 0.82 0.80 0.80	0.81

^a^ In this case, univariate model AUC.

## Data Availability

The original contributions presented in this study are included in the article/Supplementary Materials. Further inquiries can be directed to the corresponding author(s).

## Supplementary Materials

The following supporting information can be downloaded at: https://www.mdpi.com/article/10.3390/ijms26136519/s1.
