# Supplementary material for: Salivary Metabolomics Discloses Metabolite Signatures of Oral Leukoplakia with and Without Dysplasia
Source: Int J Mol Sci. 2025 Jul 7;26(13):6519. doi: 10.3390/ijms26136519 (PMC12250079; doi:10.3390/ijms26136519)
Supplement: Supplementary file 1 [file ijms-26-06519-s001.zip › ijms-3666807-supplementary.pdf]

Article

Salivary Metabolomics Discloses Metabolite Signatures of Oral Leukoplakia with and Without Dysplasia

Elena Ferrari [1,†], Rita Antonelli [2,†], Mariana Gallo [1,*], Marco Meleti [2], Giacomo Setti [3], Adele Mucci [4], Valeria Righi [5], Anna Gambini [4], Cristina Magnoni [6], Alberto Spisni [1] and Thelma A. Pertinhez [1,*]

Supplementary Material:

Figure S1: Statistical analysis of the salivary metabolite data sets from subjects with leukoplakia and dysplastic leukoplakia.

Figure S2: Heatmap of the salivary metabolite profiles derived from the leucoplakia (NDLK), dysplastic leucoplakia (DLK), and healthy control (HC) samples.

Figure S3: Multivariate ROC analysis based on selected salivary metabolites (AUC > 0.80 in univariate ROC analysis) and cross-validation.

Figure S4: Statistical performance of the OPLS-DA model of DLK+NDLK vs. HC tissues.

Table S1: PLS-DA cross-validation.

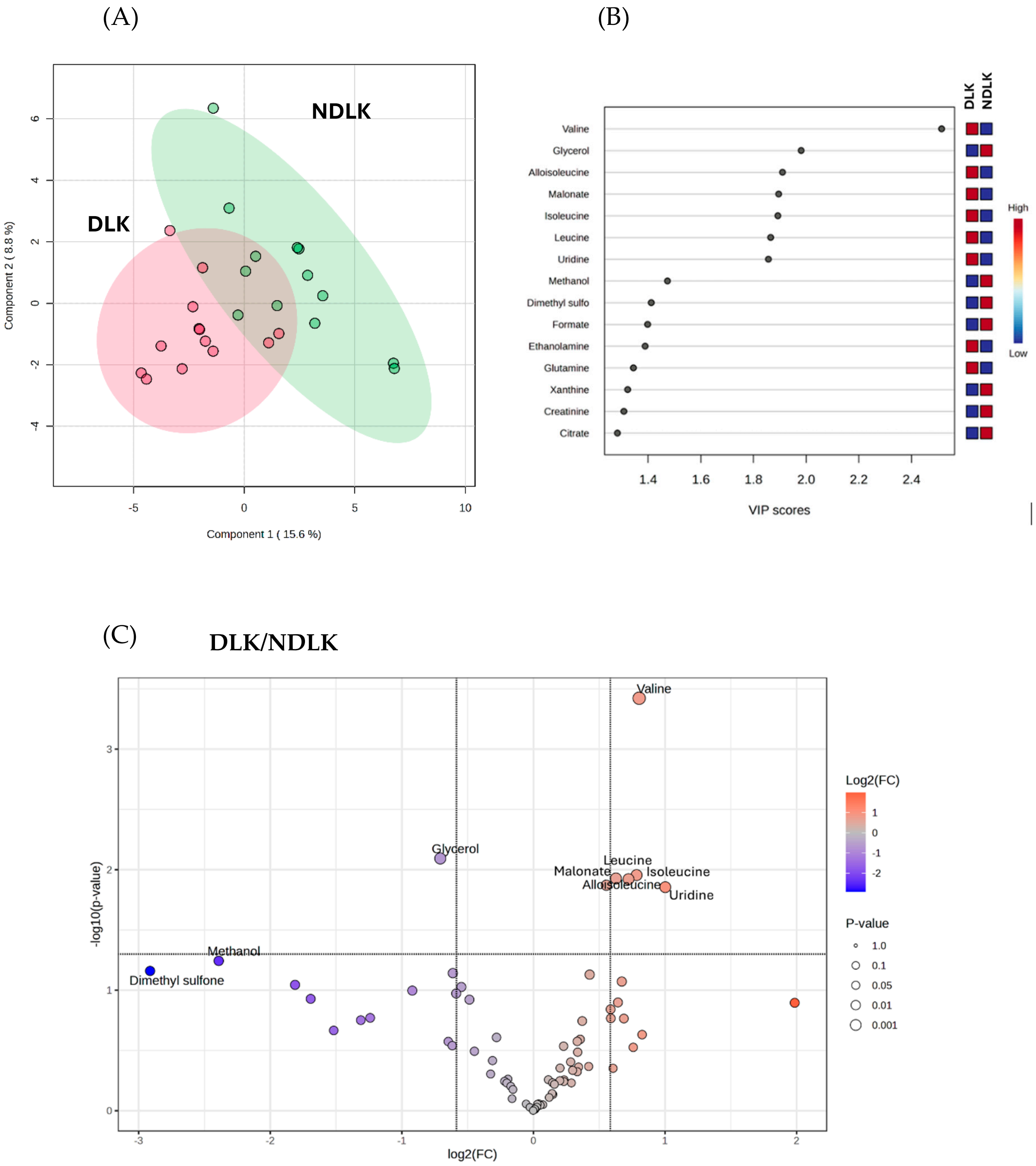

Figure S1. Statistical analysis of the salivary metabolite data sets from subjects with leukoplakia (NDLK) and dysplastic leukoplakia (DLK). (A) Partial Least Squares–Discriminant Analysis (PLS-DA) scores plot of the selected metabolomes. (B) Component 1 VIP Scores. (C) Volcano plot analysis of the DLK/NDLK metabolomes. For each metabolite compared, the plot combines its fold change ($\log_2$ (FC), on the x-axis) and p-value ($-\log_{10}$p-value, on the y-axis). Metabolites that meet the condition of $p < 0.01$ and $|FC| > 1.5$ are considered significant and are found in the upper left and right quadrants of the plot.

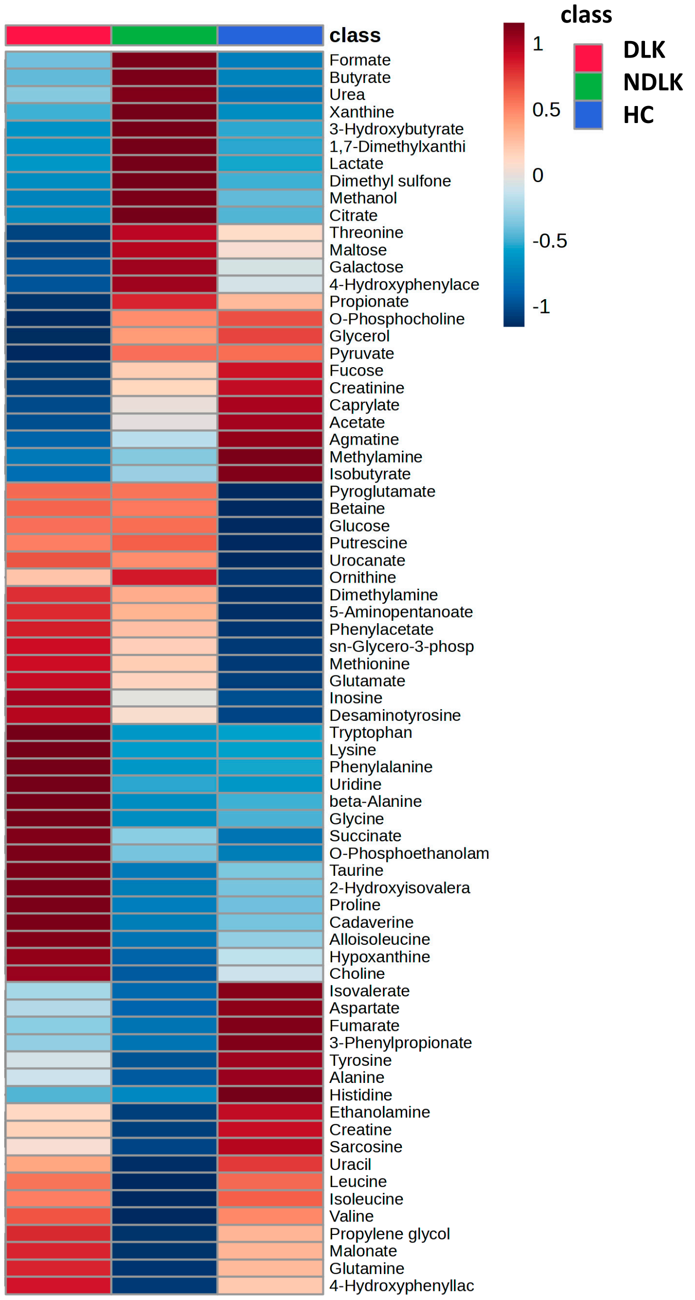

Figure S2. Heatmap of the salivary metabolite profiles derived from the leukoplakia (NDLK), dysplastic leukoplakia (DLK), and healthy control (HC) samples. The heatmap visually represents the differences in metabolite abundance across the groups. Red hues represent more concentrated metabolites, while blue hues indicate less concentrated metabolites. Each coloured cell on the map corresponds to a mean concentration value, except for the inosine metabolite, which is absent in the control group.

(A) NDLK vs HC, selected features: formate

ROC curve

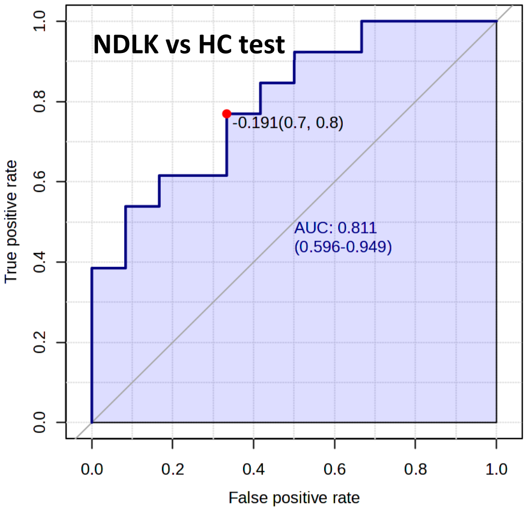

Boxplot

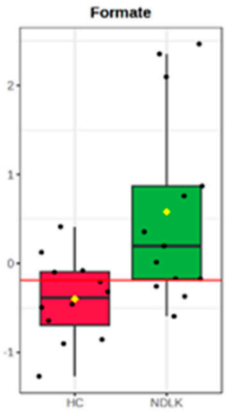

(B) DLK vs HC, selected features: sn-glycero-3-phosphocholine, urocanate, creatinine

ROC curve

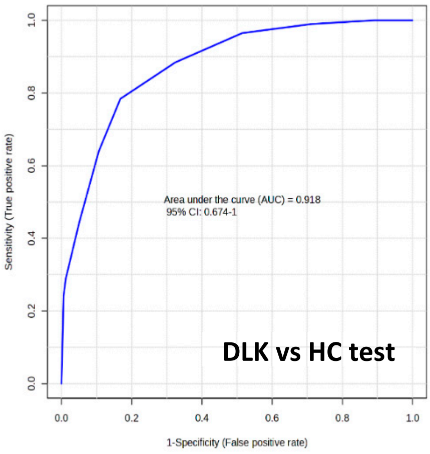

cross-validation

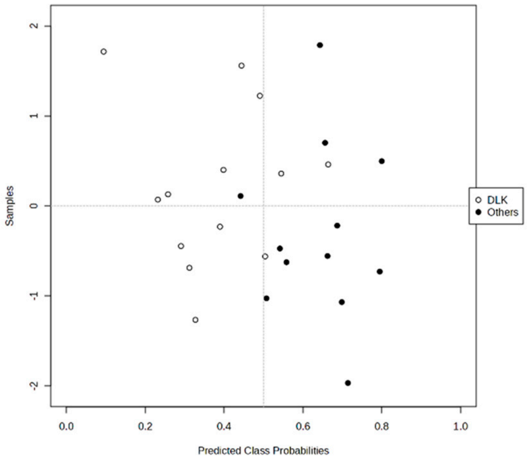

Confusion Matrix
(Cross-Validation)

	0	1
0	10	1
1	3	11

(C) DLK vs NDLK, selected features: valine, isoleucine, uridine, leucine

ROC curve

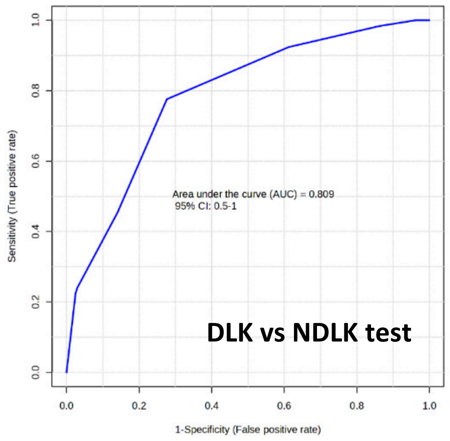

Cross-validation

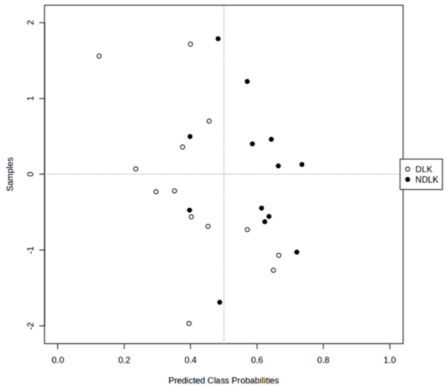

Confusion Matrix
(Cross-Validation)

	0	1
0	10	4
1	3	9

Figure S3. Multivariate ROC analysis based on selected salivary metabolites. The ROC curves (PLS-DA algorithm, two latent variables) were constructed using the indicated metabolites to evaluate their discriminative power in the diagnostic tests. Cross-validation, predicted probabilities of each sample across the 100 cross-validations. **(**A) NDLK vs HC, and the boxplot for formate variable; (B) DLK vs HC and (C) DLK vs NDLK.

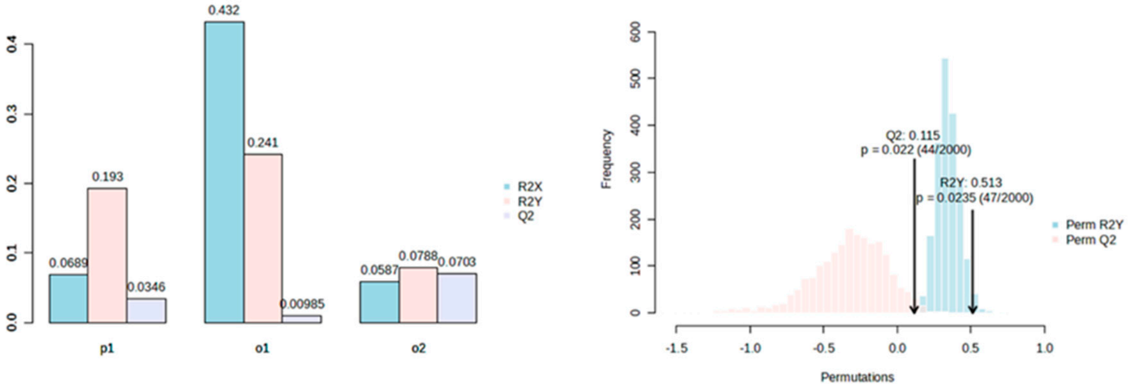

Figure S4. Statistical performance of the OPLS-DA model applied on deconvoluted ^{1}H-HR-MAS NMR CPMG signals of DLK+NDLK vs. HC tissues.

Table S1: PLS-DA cross-validation including metabolites alone and the metabolites + covariates (smoking, age, and sex).

	Metabolites	Metabolites + smoking + age + sex
**NDLK vs HC**		
accuracy	0.693	0.683
R2	0.947	0.916
Q2	0.394	0.394
**DLK vs HC**		
accuracy	0.787	0.763
R2	0.886	0.895
Q2	0.476	0.482
**DLK vs NDLK**		
accuracy	0.640	0.653
R2	0.493	0.492
Q2	0.078	0.104
